# Prognostic value of index of cardiac electrophysiological balance among US middle-aged adults

**DOI:** 10.3389/fcvm.2023.1139967

**Published:** 2023-03-22

**Authors:** Xiaolong Chen, Zhe Wang, Lin Liu, Wei Zhang, Zhiguo Tang, Bo Liu, Xuejun Zhang, Na Wei, Junkui Wang, Fuqiang Liu, Meijuan Ma

**Affiliations:** ^1^ Department of Cardiology, Shaanxi Provincial People’s Hospital, Xi’an, China; ^2^ Medical Imaging Center, Shaanxi Provincial People’s Hospital, Xi’an, China; ^3^ Department of Cardiology, The First Affiliated Hospital of Nanjing Medical University, Nanjing, China

**Keywords:** index of cardiac electrophysiological balance, electrocardiogram, all-cause mortality, prognosis biomarker, national health and nutrition examination survey

## Abstract

**Background:**

Index of cardiac electrophysiological balance (iCEB) has been widely used in clinical practice but no studies investigated the association between iCEB and prognosis in the general population.

**Objective:**

To assess the correlation between the iCEB and the prognosis in the general population.

**Methods:**

This retrospective cohort study involved adults aged 40–65 years who participated in the Third National Health and Nutrition Examination Survey (NHANES-III) and whose electrocardiograms were in sinus rhythm. The corrected iCEB (iCEBc) was the ratio of corrected QT interval (QTc) to QRS duration, and outcomes were cardiac and all-cause mortality. Cox proportional hazards regression model was used to identify the associations of iCEBc with end point. The value of iCEBc for predicting adverse events was evaluated by reclassification and discrimination analyses.

**Results:**

Among 5,010 participants (mean age 51.10 ± 7.67 years, 52.5% female), 3,454 (68.9%) were Non-Hispanic White. The mean iCEBc was 4.45 ± 0.56. A total of 2,147 deaths were recorded during a median follow-up of 319 months. The adjusted model shown iCEBc was an independent risk factor for all-cause death. The iCEBc was linearly correlated with all-cause mortality and the optimal cutoff value was 4.57 in males and 4.98 in females. In the resultant model, prolonged iCEBc remained independently associated with a higher rate of mortality (HR: 1.25; 95% CI: 1.11–1.42) and cardiac death (HR: 1.34; 95% CI: 1.04–1.71). Among the complete study population or the group with normal QTc interval, the performance of the predictive model after addition of iCEBc was not weaker than the model after the addition of prolonged QTc.

**Conclusion:**

Elevated iCEBc (male ≥4.57 and female ≥4.98) is an independent risk factor for cardiac or all-cause death among the middle-age adults. The clinical application value of iCEBc is firmly based on basic physiological principles and its application deserves further attention.

## Introduction

Sudden cardiac death due to ventricular tachyarrhythmias remains a significant problem globally (1). The tragedy of sudden, unheralded death inflicts great pain to their family and friends and incurs a large public health burden (2). Thus, early identification of the high-risk population for Sudden cardiac death is important. The electrocardiogram (ECG), as a non-invasive and easily accessible practice, can well reflect the electrical activity of the heart. Some ECG parameters, especially conduction and repolarization markers, have been used to measure the vulnerability of patients to ventricular tachycardia (VT) or ventricular fibrillation (VF) and predict the risk of sudden cardiac death (3).

Previous evidence suggested that prolonged QT interval as a measure of cardiac repolarization is associated with the occurrence of torsades depointes (TdP) and increased cardiovascular mortality (4). The QT interval is widely used clinically because of its established value in identifying the range of clinical disease ranging from electrolyte abnormalities to drug-induced cardiac toxicity to inherited channelopathies. However, if only the QT interval is assessed, those individuals prone to nontorsional VT/VF cannot be identified. In addition, the less extreme variation in QT interval length and its correlation with mortality effects in the general population remain controversial (5–7).

Drug-induced arrhythmias, rare but potentially fatal side effect, have become one of the major safety concerns of advance in the pharmaceutical industry (8). QT interval as the sole risk marker does not fully identify patients at risk of developing drug-induced arrhythmias, emphasizing the need for additional biomarkers. Index of cardiac electrophysiological balance (iCEB) was first proposed by Lu et al. in 2013 as a predictive marker for drug-induced cardiac arrhythmia (9). It is calculated by dividing the QT interval by the QRS duration (QT/QRS). This index has been proposed to be more useful than the QT interval in predicting the potential risks of drug-induced arrhythmias in rabbit models, particularly for its ability to identify long-QT related arrhythmias and non-TdP-mediated VT/VF (10). Like corrected QT interval (QTc), iCEBc is also corrected by heart rate. As the marker of assessing risk of arrhythmia, it is widely used in the clinical research, but there is still no evidence of an association between iCEBc and its prognosis (11–14).

In the light of the afore-mentioned premises, we designed this study to assess the relationship between iCEBc and all-cause or cardiac mortality in adults in the United States and explored the normal range of values for this index.

## Materials and methods

### Study design

This was a nationwide retrospective cohort study of the general population in the United States, using data from the Third National Health and Nutrition Examination Survey (NHANES-III). NHANES-III was conducted from 1988 to 1994 by the National Center for Health Statistics (NCHS) to identify the risk factors for diseases and identify important information about the healthy nutritional status of the U.S. population. The Research Ethics Review Board of NCHS approved NHANES III. The detailed survey operations manuals, consent documents and brochures of NHANES can be viewed on the website and all data related to this study are publicly available at http://www.cdc.gov/nchs/nhanes.htm.

### Study population

**Figure 1:**
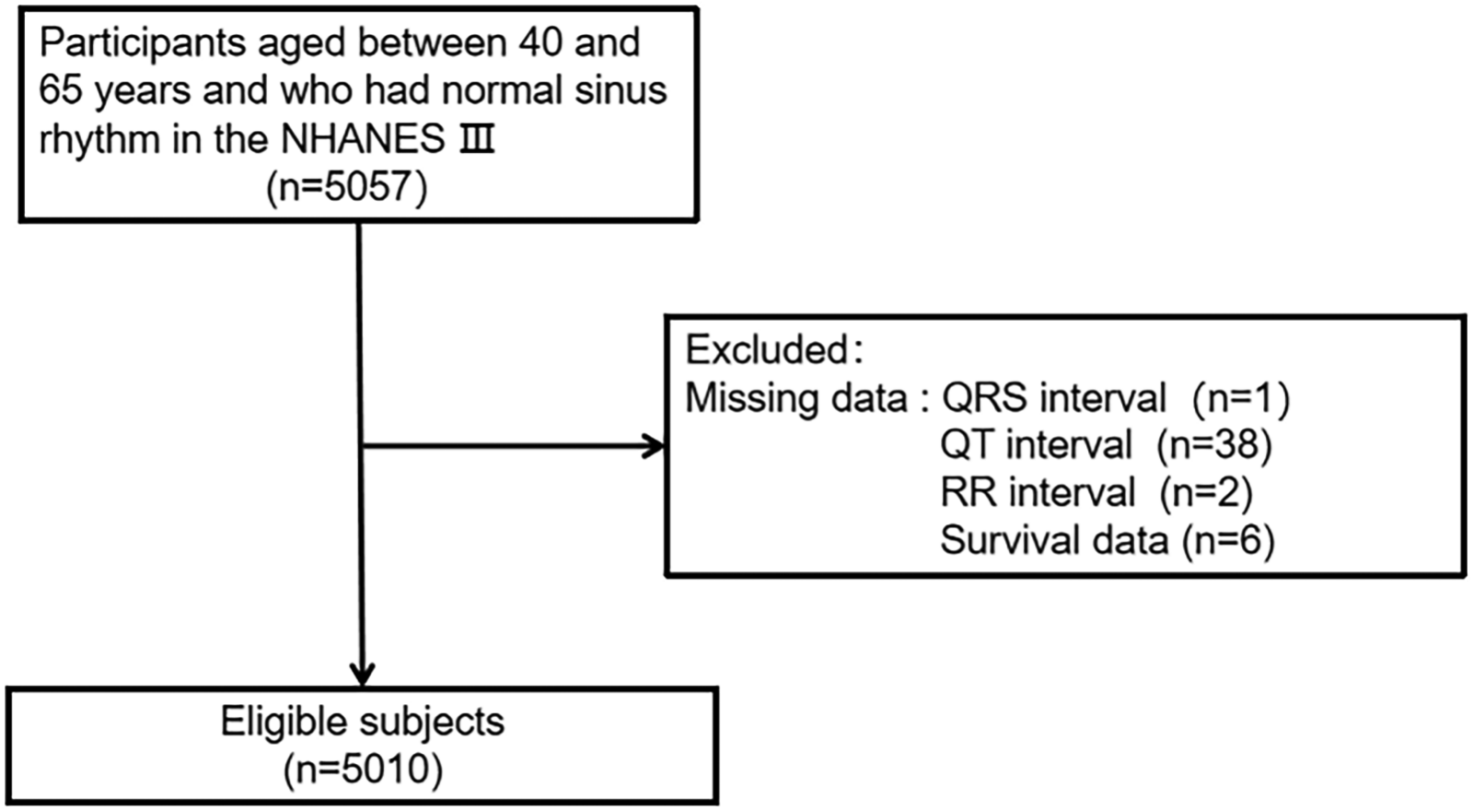
The flow chart of study case selection.

The inclusion criteria were as follows: (1) age 40–65 years; (2) ECG shown sinus rhythm. A total of 5,057 participants met the inclusion criteria. Participants who missed data were excluded (1 missing the QRS interval, 38 missing the QT interval, 2 missing the RR interval, and 6 missing survival data). Ultimately, a total of 5,010 individuals were evaluated in the current study (Figure 1).

### Electrocardiography

During the participants’ examinations, standard 12-lead ECGs were recorded by trained technicians on the Marquette MAC 12 system (Marquette Medical Systems, Inc. Milwaukee, WI). Computerized analysis of ECG data was performed using Minnesota and Novacode (15, 16). ECG was measured at rest to analyze rhythm, detect ECG abnormalities, and obtain durations and amplitudes of the ECG components. To avoid the influence of heart rate, QTc is corrected using the Bazett's formula (QTc = QT/√ RR). The iCEBc was calculated based on the ratio of QTc to QRS (QTc/QRS).

### Covariates

The data on age, sex, race/ethnicity, smoking history, and annual household income were obtained during household interviews. Body mass index (BMI) is defined as body weight (kg) divided by height (m) squared. The history of some diseases, including myocardial infarction, stroke, heart failure, and diabetes, was obtained through self-reporting. In addition, family history of cardiovascular disease and the use of antihypertensive drugs were obtained by self-reported form. Blood pressure measurements were the average of six measurements (three in-home measurements and three in mobile center measurements). Total serum cholesterol and high-density lipoprotein cholesterol (HDL-c) levels were measured enzymatically. Serum potassium and calcium levels were assessed to exclude their roles in the development of arrhythmias.

### Ascertainment of mortality outcomes

The primary outcome of this study was all-cause mortality. Mortality data were obtained by linking to the National Death Index, a publicly available dataset in the United States as of April 2022. The database was used to determine the mortality status of eligible participants. According to the tenth revision of the International Classification of Diseases (ICD-10), data on potential causes of death are used for case definition. Cardiac death was defined as death due to heart disease (ICD-10 codes I00–I09, I11, I13 and I20–I51).

### Statistical analyses

Descriptive statistics were used to summarize baseline data, including the mean and standard deviation of normally distributed continuous data, and the percentage of categorical data.

The Cox proportional hazards regression model was used to estimate the hazard ratios (HRs) and 95% confidence interval (CI) associated with cardiovascular and all-cause mortality and iCEBc (continuous variable). Multivariable models were adjusted for all available variables based on clinical experience or previous studies (17, 18). We evaluated multicollinearity in the final model using the variance inflation factor (VIF) test. Restricted cubic splines (RCS) were used to determine the linear relationship between iCEBc and death. Because of known differences in the QT interval, RCS was performed separately in males and females.

Use Log-rank analysis and the “cutoff” package (R version. 4.1.3) to find the valid cutoff values for iCEBc (continuous variable). We found that the optimal cut-off values for iCEBc were 4.57 for males and 4.98 for females. Therefore, iCEBc was converted to dichotomous variables according to cut-off values (normal iCEBc: male <4.57, female <4.98; prolonged iCEBc: male ≥4.57, female ≥4.98).

In addition, we repeated the Cox regression analysis, adjusting for the same confounders in the above model. Subgroup analyses were performed for sex, age, race, prolonged QTc (male >450 ms, female >460 ms) and intraventricular conduction delays (bundle branch blocks or QRS > 120 ms). *P* for interaction values were calculated separately.

The addition of iCEBc (continuous value) and prolonged QTc interval to the traditional model were compared for improvements in risk prediction of adverse outcomes. We calculated Area Under Curve (AUC), Integrated Discrimination Index (IDI) and Net Rclassification Improvement (NRI) every 12 months. The random seed was set to 0403 and the number of iterations when calculating NRI and IDI was set to 99. Missing values were filled using multiple imputation. All data management and statistical analysis were conducted in R version 4.1.3. and *P* value less than 0.05 was considered significant.

## Results

### Characteristics of study population

The baseline characteristics of the total population are presented in Table 1. Among 5,010 participants from NHANES III (mean age 51.10 ± 7.67 years), 2,629 (52.5%) were females and 3,454 (68.9%) were Non-Hispanic White. The mean iCEBc was 4.45 ± 0.56. During the median follow-up of 319 months, a total of 2,147 deaths were recorded. The iCEBc was significantly higher for female non-survivors compared with female survivors (*P *= 0.001). Similar results were observed in males.

**Table 1:** Baseline characteristics of US participants.

Variables	Male			Female		
	Alive	Dead	*P* value	Alive	Dead	*P* value
	*N* = 1,236	*N* = 1,145		*N* = 1,627	*N* = 1,002	
Age	48.09 ± 6.75	54.53 ± 7.30	<0.001	48.51 ± 6.75	54.85 ± 7.35	<0.001
Race/ethnicity, %			0.002			0.029
White	878 (71.0)	795 (69.4)		1,112 (68.3)	669 (66.8)	
Black	303 (24.5)	325 (28.4)		456 (28.0)	312 (31.1)	
Others	55 (4.4)	25 (2.2)		59 (3.6)	21 (2.1)	
Total annual income <$20,000, %	350 (28.6)	504 (44.8)	<0.001	560 (35.1)	514 (52.5)	<0.001
Smoke, %			<0.001			<0.001
Never	429 (34.7)	226 (19.7)		1,021 (62.8)	449 (44.8)	
Former	470 (38.0)	431 (37.6)		331 (20.3)	212 (21.2)	
Current	337 (27.3)	488 (42.6)		275 (16.9)	341 (34.0)	
Body mass index^a^, kg/m^2^	27.45 ± 4.26	27.73 ± 5.15	0.186	28.28 ± 6.05	29.49 ± 6.90	<0.001
Myocardial infarction, %	26 (2.1)	110 (9.7)	<0.001	20 (1.2)	44 (4.4)	<0.001
Stroke, %	8 (0.6)	41 (3.6)	<0.001	11 (0.7)	32 (3.2)	<0.001
Congestive heart failure, %	13 (1.1)	70 (6.1)	<0.001	22 (1.4)	43 (4.3)	<0.001
Diabetes mellitus, %	44 (3.6)	162 (14.2)	<0.001	99 (6.1)	184 (18.4)	<0.001
Family history of cardiovascular disease, %	158 (12.9)	154 (13.7)	0.649	245 (15.3)	175 (17.7)	0.109
Antihypertensive medications, %	190 (15.5)	327 (28.7)	<0.001	294 (18.1)	377 (37.6)	<0.001
HDL-c cholesterol, mg/dl	45.35 ± 14.07	46.06 ± 15.22	0.242	55.72 ± 15.65	53.28 ± 16.58	<0.001
Total cholesterol, mg/dl	212.02 ± 40.03	215.24 ± 43.47	0.063	212.96 ± 41.26	225.02 ± 47.22	<0.001
Diastolic blood pressure, mmHg	80.63 ± 10.04	81.89 ± 12.35	0.007	76.90 ± 10.19	78.65 ± 12.00	<0.001
Systolic blood pressure, mmHg	125.67 ± 14.53	135.36 ± 19.06	<0.001	123.77 ± 17.30	134.10 ± 21.46	<0.001
Serum potassium, mmol/L	4.05 ± 0.31	4.08 ± 0.34	0.025	3.99 ± 0.29	4.00 ± 0.35	0.352
Serum calcium, mmol/L	2.30 ± 0.11	2.29 ± 0.12	0.195	2.30 ± 0.11	2.31 ± 0.12	0.030
QRS duration, msec	101.11 ± 11.59	101.97 ± 13.21	0.089	94.03 ± 10.30	94.71 ± 12.46	0.127
QT interval, msec	400.16 ± 28.37	402.04 ± 31.50	0.125	407.87 ± 27.63	406.17 ± 32.67	0.153
iCEBc	4.18 ± 0.46	4.25 ± 0.51	<0.001	4.66 ± 0.50	4.72 ± 0.56	0.001
Corrected QT interval, msec	417.50 ± 20.88	427.27 ± 23.55	<0.001	433.32 ± 21.64	441.15 ± 22.47	<0.001
RR interval, msec	0.93 ± 0.15	0.90 ± 0.16	<0.001	0.89 ± 0.13	0.86 ± 0.14	<0.001

Values are mean ± SD or *n* (%). HDL-c, high-density lipoprotein cholesterol; iCEB, index of cardiac electrophysiological balance; iCEBc, corrected index of cardiac electrophysiological balance.

^a^ Body mass index is defined as body weight (kg) divided by height (m) squared.

### The associations between iCEBc and all-cause or cardiac death

During the follow-up time of 373 months, we found a 18% increased risk of death for each additional standard deviation of iCEBc (HR: 1.18; 95% CI: 1.09–1.29; Model 2: adjusted for age, sex, race/ethnicity, income, and BMI). In the full model after multi-factor adjustment (Model 4: adjusted for age, sex, race/ethnicity, income, body mass index, smoker, myocardial infarction, stroke, heart failure, diabetes mellitus, family history of cardiovascular disease, antihypertensive medications use, HDL-c, total cholesterol, diastolic blood pressure, systolic blood pressure, serum potassium, serum calcium, QRS duration, prolonged QTc and RR interval), we observed that iCEBc was an independent risk factor for all-cause death (HR: 1.61; 95% CI: 1.30–2.01). Meanwhile, iCEBc was also an independent risk factor for cardiac death (HR: 1.85; 95% CI: 1.20–2.87). All results were presented in Table 2. The variables added to the adjusted model all had VIF values less than 3, and there was no collinearity between the variables.

**Table 2:** Hazard ratios for the association between iCEBc and All-cause death and cardiac death.

Death	iCEBc^a^	Model 1^b^		Model 2^c^		Model 3^d^		Model 4^e^	
		HR (95% CI)	*P-*value	HR (95% CI)	*P-*value	HR (95% CI)	*P-*value	HR (95% CI)	*P-*value
All-cause	Per-SD increase	1.06 (0.98–1.15)	0.127	1.18 (1.09–1.29)	<0.001	1.13 (1.04–1.23)	0.003	1.61 (1.30–2.01)	<0.001
	Normal iCEBc	1.00 (reference)		1.00 (reference)		1.00 (reference)		1.00 (reference)	
	Prolonged iCEBc^f^	1.36 (1.24–1.49)	<0.001	1.32 (1.20–1.45)	<0.001	1.24 (1.13–1.36)	<0.001	1.25 (1.11–1.42)	<0.001
Cardiac	Per-SD increase	1.06 (0.91–1.24)	0.456	1.24 (1.05–1.47)	0.013	1.19 (1.01–1.41)	0.038	1.85 (1.20–2.87)	0.006
	Normal iCEBc	1.00 (reference)		1.00 (reference)		1.00 (reference)		1.00 (reference)	
	Prolonged iCEBc	1.44 (1.19–1.73)	<0.001	1.41 (1.17–1.70)	<0.001	1.33 (1.10–1.61)	0.003	1.34 (1.04–1.71)	0.022

^a^ iCEBc, index of cardiac electrophysiological balance with heart rate correction.

^b^ Model 1: unadjusted model.

^c^ Model 2: adjusted for age, sex, race/ethnicity, income, body mass index.

^d^ Model 3: Model 2 additionally adjusted for smoke, myocardial infarction, stroke, congestive heart failure, diabetes mellitus, family history of cardiovascular disease, antihypertensive medications use, HDL cholesterol, total cholesterol, diastolic blood pressure, systolic blood pressure, serum potassium, serum calcium.

^e^ Model 4: Model 3 additionally adjusted for QRS duration, corrected QT interval and RR interval.

^f^ Prolonged iCEBc: male ≥4.57, female ≥4.98.

**Figure 2:**
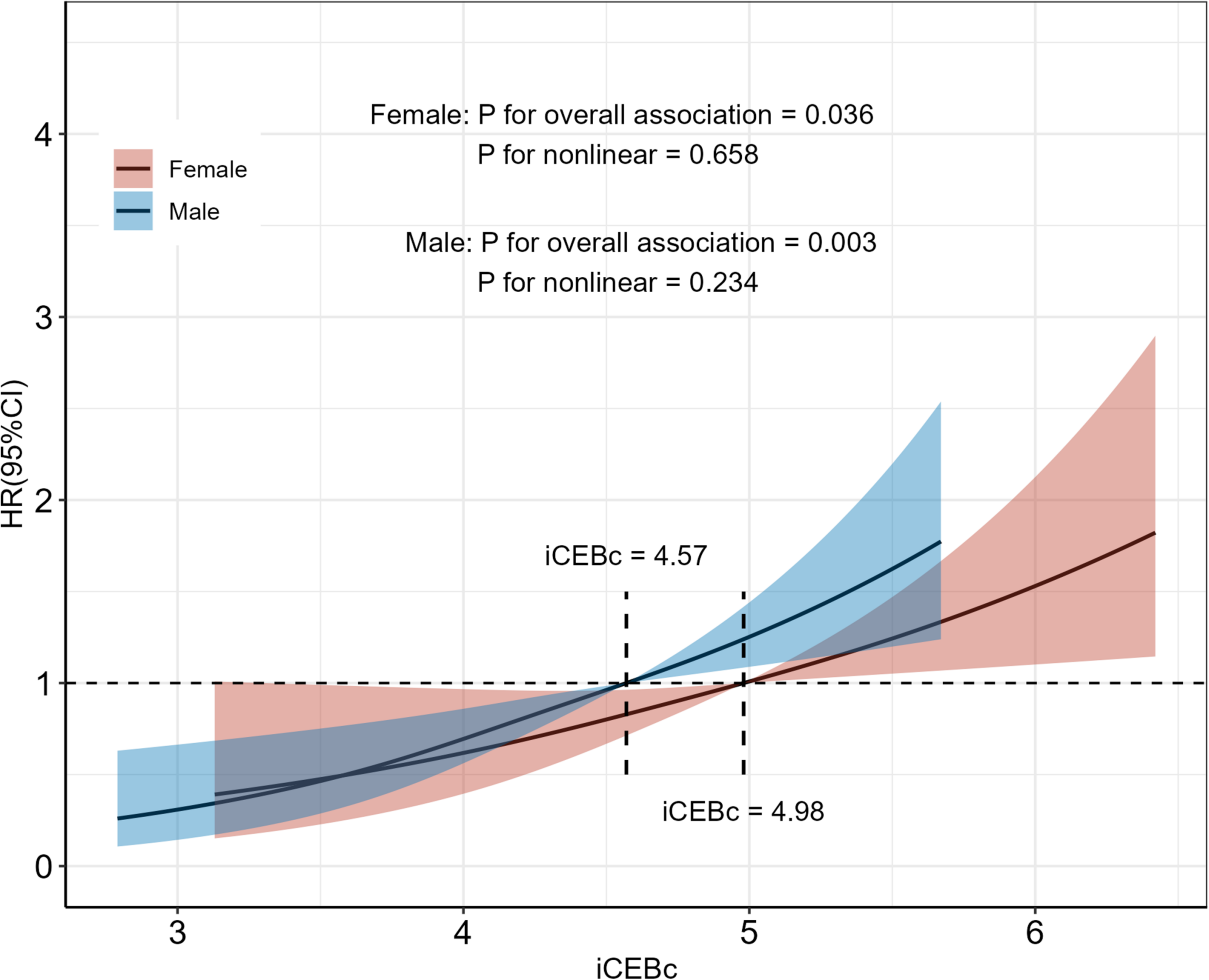
Restricted cubic spline of the association between iCEBc and All-cause death. Participants were divided into two groups according to the iCEBc cut-off value (male: 4.57, female: 4.98). HR, hazard ratio; 95% CI, 95% confidence interval.

**Figure 3:**
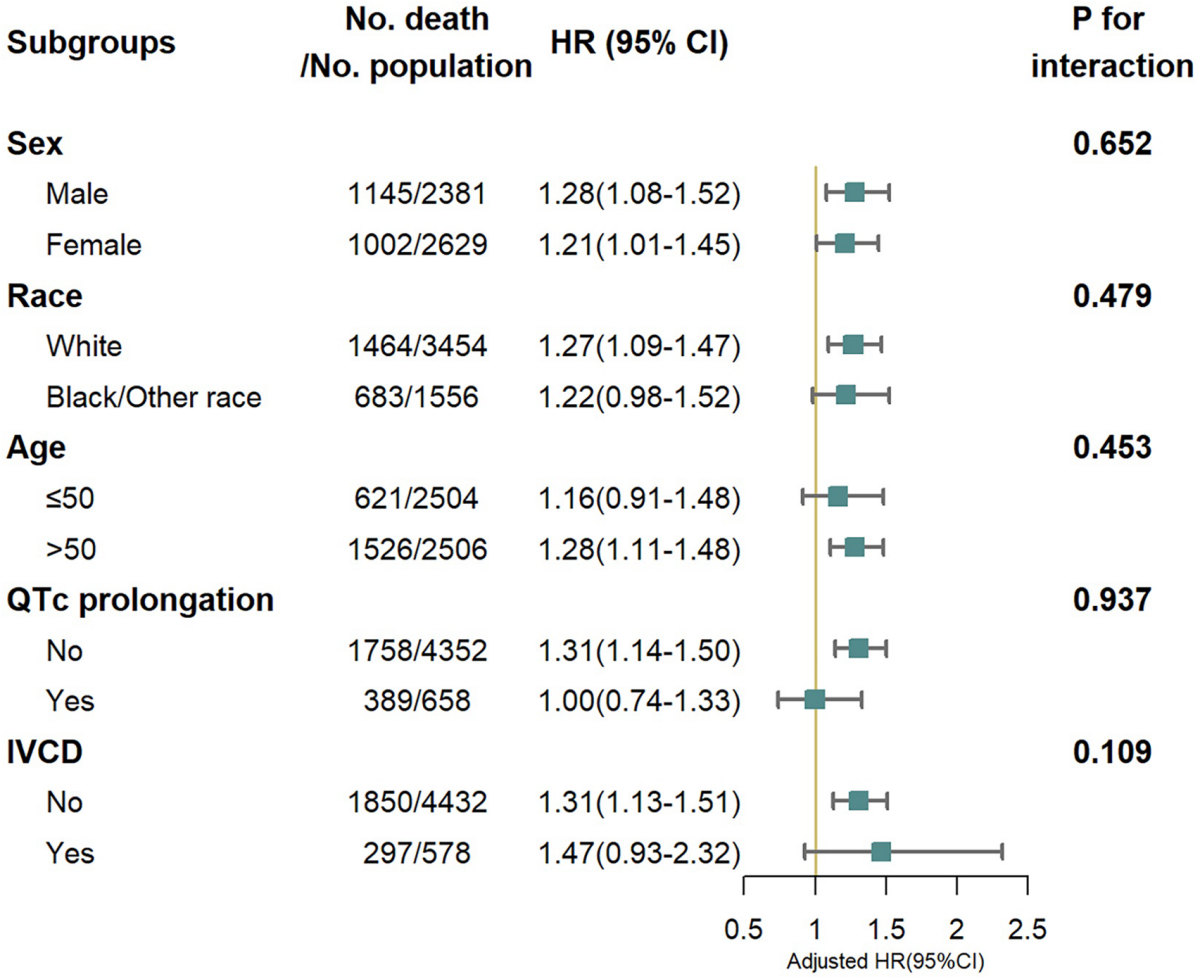
All-cause mortality risk in subgroup analysis. IVCD, Intraventricular conduction delay (bundle branch blocks or QRS > 120 ms). Adjusted for age, sex, race/ethnicity, income, body mass index, smoke, myocardial infarction, stroke, congestive heart failure, diabetes mellitus, family history of cardiovascular disease, antihypertensive medications use, HDL cholesterol, total cholesterol, diastolic blood pressure, systolic blood pressure, serum potassium, serum calcium, QRS duration, corrected QT interval and RR interval.

We also found a linear relationship between iCEBc and all-cause mortality in either the male or female group (Figure 2). Therefore, participants were divided into two groups according to the cutoff value (male: 4.57, female: 4.98). After converting continuous iCEBc to categorical variables (normal iCEBc and prolonged iCEBc), the resultant model (Model 4) showed that prolonged iCEBc remained independently associated with a higher ratio of all-cause mortality (HR: 1.25; 95% CI: 1.11–1.42) and cardiac death (HR: 1.34; 95% CI: 1.04–1.71) (Table 2). In addition, we tested the interaction of a number of variables with iCEBc (including sex, age, ethnicity, prolonged QTc, and intraventricular conduction delay). However, we did not find any significant interactions between them (Figure 3).

### Reclassification and discrimination statistics for all-cause death

**Figure 4:**
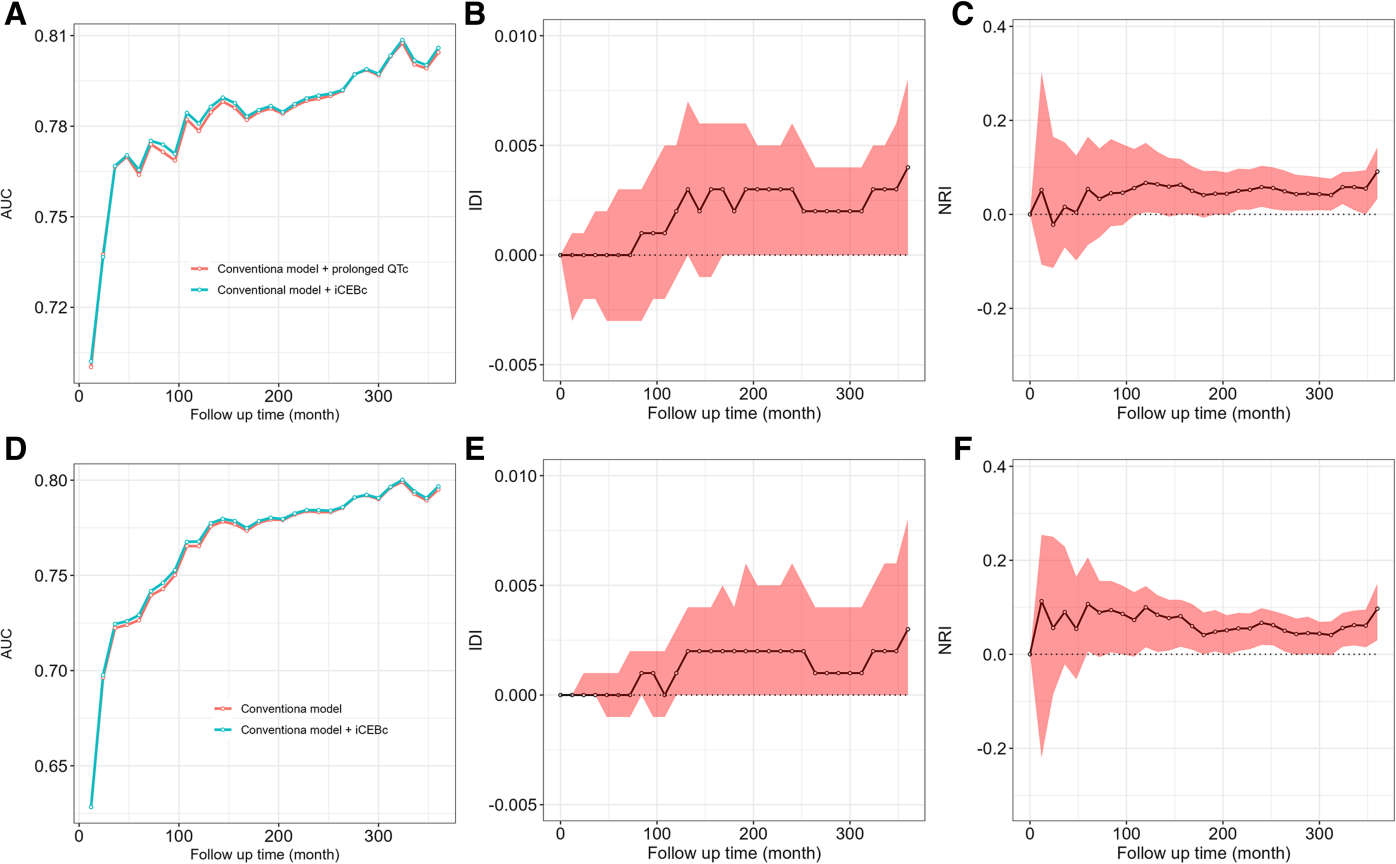
The comparison of AUC, IDI, NRI between conventional model adding iCEBc and conventional model adding prolonged QTc interval. **A–C** showed the complete study population. **D–F** showed the group with normal QTc interval. AUC, area under curve; IDI, integrated discrimination index; NRI, net reclassification improvement.

The AUC, IDI and NRI were calculated to evaluate whether adding iCEBc to the conventional model could improve prediction performance compared with adding prolonged QTc to the conventional model. The results were shown in Figure 4, Supplementary Tables S1, S2.

In the complete study population or the group with normal QTc interval, we found that the model after the addition of iCEBc was not weaker than the model after the addition of prolonged QTc by comparing the AUC, IDI and NRI. The IDI and NRI values suggested that the model after adding iCEBc did not have a significant improvement in predicting the primary outcome over 10 years compared to models including prolonged QTc. However, when we extended the follow-up time, the performance of the model improved significantly after adding iCEBc in the whole analyzed population. NRI (0.058, 95% CI: 0.016–0.103; *P* < 0.05) and IDI (0.003, 95% CI: 0.000–0.006; *P* < 0.05) peaked at 240 months (Supplementary Table S1). Among the subgroup with normal QTc interval, the NRI at the mean event rate showed significant reclassification of participant risk by addition of iCEBc at multiple time points after 5 years.

## Discussion

Our data showed strong clinical evidence of the prognostic significance of elevated iCEBc of ECG in middle-age adults with sinus rhythm. Prolonged iCEBc was associated with an increased risk of all-cause or cardiac mortality and the association was independent of conventional risk factors, even QRS duration and prolonged QTc.

iCEB is considered a surrogate marker of the cardiac wavelength *λ* (*λ *= effective refractory period × conduction velocity), and is a manifestation of the balance between cardiac repolarization and depolarization (9, 19). In rabbit models of myocardial infarction with delayed left ventricular systolic dysfunction, extreme iCEB reduction might contribute to preclinical risk assessment of severe drug-induced arrhythmias (20). Increased iCEB may lead to TdP-like ventricular arrhythmias, while decreased iCEB may result in non-TdP-mediated arrhythmogenic behavior (9). Its physiologic basis provides valuable clinical information, and as such, this index has been applied to clinical research as a VT risk assessment tool. For example, iCEB and iCEBc were both increased in people with slow coronary blood flow (iCEB: 4.90 ± 0.40 vs. 4.20 ± 0.40, *P *< 0.001; iCEBc: 5.70 ± 0.30 vs. 4.40 ± 0.30, *P *< 0.001) (14). Another cross-sectional study showed that smokers had elevated iCEBc and prolonged QTc compared with non-smokers (iCEBc: 5.10 ± 0.49 vs. 4.68 ± 0.39, *P *< 0.001) (21). In some respects, iCEBc is more sensitive to clinical risk factors than QTc. One study showed that iCEB and iCEBc were elevated after hemodialysis (iCEB: 4.38 ± 0.76 vs. 4.02 ± 0.73, *P *= 0.016; iCEBc: 4.91 ± 0.73 vs. 4.42 ± 0.80, *P *= 0.006), but there was no significant change in the QT interval or QTc, indicating that the new ECG marker was more sensitive to cardiac electrophysiological balance (22). However, in some studies, differences in iCEB were often explained by differences in QTc or QRS duration (12, 23). In fact, no study has directly linked iCEBc to cardiac or all-cause death. Our research is the first to demonstrate a significant linear correlation between iCEBc and death, and that this correlation was independent of QTc and QRS duration.

We noticed significant differences in QRS duration and QT interval between males and females, as previously mentioned in the other studies (24–26). These differences may be the result of complex interaction between sex steroids and gonadotropins (27). Based on this, we decided to calculate the cutoff values for iCEBc separately in males and females. The robustness of cutoff values was validated in subgroup analysis of sex. Subgroup analyses also demonstrated statistical evidence of heterogeneity which might be due to the smaller sample size and lower event rates in some specific subgroups. Simultaneously, there was no interaction between iCEBc and these grouping variables, so the relationship between iCEBc and all-cause mortality can be considered robust. In the normal QTc subgroup of interest, prolonged iCEBc remained significantly associated with all-cause mortality. This suggests that the indicator can identify high-risk populations that QTc cannot identify. Our further analysis confirmed this view that the predictive value of iCEBc in identifying patients with poor outcome was no weaker than prolonged QTc. In the long term, it can even compensate for the defects of QTc. Both iCEBc and prolonged QTc are important indicators of ventricular arrhythmias and are both associated with adverse outcome (28, 29). Therefore, we compared iCEBc with prolonged QTc to investigate whether adding iCEBc to traditional risk prediction model improved the prediction of adverse outcomes compared with adding prolonged QTc to traditional risk-prediction model. We used AUC, NRI and IDI to quantify the improvement of prediction. Given the time-dependent effect in the Cox model, we evaluated two models separately at follow-up time points from 0 to 360 months. According to the NRI and IDI, when iCEBc was added to the full study population using traditional models, more individuals were more correctly reclassified. It can be seen that the ability of the model adding iCEBc to identify high-risk group was not weaker than that of the model after adding prolonged QTc. In the long term, iCEBc seems to have some advantages. Moreover, iCEBc inproved the predictive ability of traditional model in normal QTc subpopulations at certain time points, demonstrating that iCEBc has great potential for clinical application in high-risk patients who are difficult to identify QTc. Therefore, it may address concerns about using only the QT interval as a drug-induced arrhythmia risk marker, alleviate the dilemma of arrhythmia prediction (30, 31), and help clinicians and patients make optimal decisions to minimize the risk of using arrhythmogenic medications (32).

The QT interval is a marker of both ventricular depolarization and repolarization, mainly repolarization, while the new biomarker emphasizes the balance of the two phases. For this reason, the interpretation of iCEBc difference should not be limited to change in QTc or QRS, and need to be considered comprehensively in future research and practice.

There are some limitations that should be discussed. First, the database cannot provide information about the exact arrhythmia. Therefore, we cannot directly assess the association between iCEBc and VT occurrence. However, we comprehensively assessed the risk of all-cause death and cardiovascular death. Second, this study was only a survey of the general middle-aged population. Future studies should be conducted in larger populations and should compare VT measurements between normal and susceptible populations.

## Conclusion

Elevated iCEBc (male ≥4.57, female ≥4.98) is an independent risk factor for cardiac or all-cause death among middle-age adults. The clinical application value of iCEBc is based on its basic physiological principles and its clinical application deserves further attention.

## Data Availability

The raw data supporting the conclusions of this article will be made available by the authors, without undue reservation.
