# Supplementary material for: Prognostic value of index of cardiac electrophysiological balance among US middle-aged adults
Source: Front Cardiovasc Med. 2023 Mar 22;10:1139967. doi: 10.3389/fcvm.2023.1139967 (PMC10073598; doi:10.3389/fcvm.2023.1139967)
Supplement: Supplementary file 1 [file Datasheet1.docx]

**SUPPLEMENTAL MATERIAL**

This appendix has been provided by the authors to give readers additional information about their work.

**Prognostic Value of Index of Cardiac Electrophysiological Balance Among US Middle-aged Adults**

Xiaolong Chen^1,2†^, Zhe Wang^3†^, Lin Liu^1^, Wei Zhang^1^, Zhiguo Tang^1^, Bo Liu^1^, Xuejun Zhang^1^, Na Wei^1^, Junkui Wang^1^, Fuqiang Liu^1^, Meijuan Ma^1*^

†These two authors contributed equally to this work.

^1^Department of Cardiology, Shaanxi Provincial People’s Hospital, Xi’an, China.

^2^Medical Imaging Center, Shaanxi Provincial People’s Hospital, Xi’an, China.

^3^Department of Cardiology, The First Affiliated Hospital of Nanjing Medical University, Nanjing, China.

**Supplementary Table 1. AUC, IDI, and NRI were Calculated in the Whole Population**

| Times (month) | Total study population | | | | | |
| --- | --- | --- | --- | --- | --- | --- |
|  | AUC | | IDI | | NRI | |
|  | Conventiona model + prolonged QTc | Conventiona model + iCEBc | Conventiona model + prolonged QTc | Conventiona model + iCEBc | Conventiona model + prolonged QTc | Conventiona model + iCEBc |
|  |  |  |  |  |  |  |
| 12 | 0.70 (0.59-0.81) | 0.70 (0.60-0.81) | ref | 0.000 (-0.003-0.001) | ref | 0.052 (-0.107-0.302) |
| 24 | 0.74 (0.68-0.80) | 0.74 (0.67-0.80) | ref | 0.000 (-0.002-0.001) | ref | -0.022 (-0.114-0.165) |
| 36 | 0.77 (0.72-0.81) | 0.77 (0.72-0.81) | ref | 0.000 (-0.002-0.002) | ref | 0.016 (-0.070-0.153) |
| 48 | 0.77 (0.73-0.81) | 0.77 (0.73-0.81) | ref | 0.000 (-0.003-0.002) | ref | 0.004 (-0.098-0.125) |
| 60 | 0.76 (0.73-0.80) | 0.77 (0.73-0.80) | ref | 0.000 (-0.003-0.003) | ref | 0.054 (-0.065-0.165) |
| 72 | 0.77 (0.74-0.81) | 0.78 (0.74-0.81) | ref | 0.000 (-0.003-0.003) | ref | 0.033 (-0.049-0.140) |
| 84 | 0.77 (0.74-0.80) | 0.77 (0.74-0.80) | ref | 0.001 (-0.003-0.003) | ref | 0.045 (-0.025-0.160) |
| 96 | 0.77 (0.74-0.80) | 0.77 (0.74-0.80) | ref | 0.001 (-0.002-0.004) | ref | 0.046 (-0.023-0.149) |
| 108 | 0.78 (0.76-0.81) | 0.78 (0.76-0.81) | ref | 0.001 (-0.002-0.005) | ref | 0.056 (-0.002-0.139) |
| 120 | 0.78 (0.75-0.80) | 0.78 (0.76-0.80) | ref | 0.002 (-0.001-0.005) | ref | 0.067 (0.004-0.152) |
| 132 | 0.78 (0.76-0.81) | 0.79 (0.77-0.81) | ref | 0.003 (0.000-0.007) | ref | 0.064 (0.003-0.133) |
| 144 | 0.79 (0.77-0.81) | 0.79 (0.77-0.81) | ref | 0.002 (-0.001-0.006) | ref | 0.059 (-0.005-0.120) |
| 156 | 0.79 (0.77-0.81) | 0.79 (0.77-0.81) | ref | 0.003 (-0.001-0.006) | ref | 0.063 (0.000-0.118) |
| 168 | 0.78 (0.76-0.80) | 0.78 (0.76-0.80) | ref | 0.003 (0.000-0.006) | ref | 0.050 (0.000-0.102) |
| 180 | 0.78 (0.77-0.80) | 0.79 (0.77-0.80) | ref | 0.002 (0.000-0.006) | ref | 0.041 (-0.007-0.092) |
| 192 | 0.79 (0.77-0.80) | 0.79 (0.77-0.80) | ref | 0.003 (0.000-0.006) | ref | 0.044 (0.000-0.093) |
| 204 | 0.78 (0.77-0.80) | 0.78 (0.77-0.80) | ref | 0.003 (0.000-0.005) | ref | 0.044 (-0.001-0.089) |
| 216 | 0.79 (0.77-0.80) | 0.79 (0.77-0.80) | ref | 0.003 (0.000-0.005) | ref | 0.050 (0.010-0.097) |
| 228 | 0.79 (0.77-0.80) | 0.79 (0.77-0.80) | ref | 0.003 (0.000-0.005) | ref | 0.052 (0.010-0.096) |
| 240 | 0.79 (0.77-0.80) | 0.79 (0.78-0.81) | ref | 0.003 (0.000-0.006) | ref | 0.058 (0.016-0.103) |
| 252 | 0.79 (0.78-0.80) | 0.79 (0.78-0.81) | ref | 0.002 (0.000-0.005) | ref | 0.056 (0.011-0.100) |
| 264 | 0.79 (0.78-0.81) | 0.79 (0.78-0.81) | ref | 0.002 (0.000-0.004) | ref | 0.049 (0.008-0.093) |
| 276 | 0.80 (0.78-0.81) | 0.80 (0.78-0.81) | ref | 0.002 (0.000-0.004) | ref | 0.043 (0.008-0.084) |
| 288 | 0.80 (0.79-0.81) | 0.80 (0.79-0.81) | ref | 0.002 (0.000-0.004) | ref | 0.044 (0.008-0.082) |
| 300 | 0.80 (0.78-0.81) | 0.80 (0.78-0.81) | ref | 0.002 (0.000-0.004) | ref | 0.043 (0.009-0.079) |
| 312 | 0.80 (0.79-0.82) | 0.80 (0.79-0.82) | ref | 0.002 (0.000-0.004) | ref | 0.041 (0.008-0.076) |
| 324 | 0.81 (0.79-0.82) | 0.81 (0.80-0.82) | ref | 0.003 (0.000-0.005) | ref | 0.058 (0.022-0.090) |
| 336 | 0.80 (0.79-0.81) | 0.80 (0.79-0.82) | ref | 0.003 (0.000-0.005) | ref | 0.058 (0.009-0.091) |
| 348 | 0.80 (0.78-0.82) | 0.80 (0.78-0.82) | ref | 0.003 (0.000-0.006) | ref | 0.055 (0.000-0.094) |
| 360 | 0.70 (0.59-0.81) | 0.70 (0.60-0.81) | ref | 0.004 (0.000-0.008) | ref | 0.091 (0.033-0.142) |

**Supplementary Table 2. AUC, IDI, and NRI were Calculated in the Population with Normal QTc Interval**

| Times (month) | Population with normal QTc interval | | | | | |
| --- | --- | --- | --- | --- | --- | --- |
|  | AUC | | IDI | | NRI | |
|  | Conventiona model | Conventiona model + iCEBc | Conventiona model | Conventiona model + iCEBc | Conventiona model | Conventiona model + iCEBc |
|  |  |  |  |  |  |  |
| 12 | 0.63 (0.51-0.75) | 0.63 (0.51-0.75) | ref | 0.000 (0.000-0.000) | ref | 0.113 (-0.220-0.254) |
| 24 | 0.70 (0.62-0.77) | 0.70 (0.62-0.77) | ref | 0.000 (0.000-0.001) | ref | 0.056 (-0.085-0.25) |
| 36 | 0.72 (0.66-0.78) | 0.72 (0.67-0.78) | ref | 0.000 (0.000-0.001) | ref | 0.090 (-0.021-0.229) |
| 48 | 0.72 (0.67-0.78) | 0.73 (0.67-0.78) | ref | 0.000 (-0.001-0.001) | ref | 0.054 (-0.053-0.165) |
| 60 | 0.73 (0.68-0.77) | 0.73 (0.68-0.77) | ref | 0.000 (-0.001-0.001) | ref | 0.107 (0.006-0.206) |
| 72 | 0.74 (0.70-0.78) | 0.74 (0.70-0.78) | ref | 0.000 (-0.001-0.002) | ref | 0.089 (-0.006-0.156) |
| 84 | 0.74 (0.71-0.78) | 0.75 (0.71-0.78) | ref | 0.001 (0.000-0.002) | ref | 0.094 (0.004-0.156) |
| 96 | 0.75 (0.72-0.78) | 0.75 (0.72-0.79) | ref | 0.001 (-0.001-0.002) | ref | 0.086 (0.000-0.146) |
| 108 | 0.77 (0.73-0.80) | 0.77 (0.74-0.80) | ref | 0.000 (-0.001-0.002) | ref | 0.073 (-0.005-0.132) |
| 120 | 0.77 (0.74-0.79) | 0.77 (0.74-0.80) | ref | 0.001 (0.000-0.003) | ref | 0.100 (0.015-0.145) |
| 132 | 0.78 (0.75-0.80) | 0.78 (0.75-0.80) | ref | 0.002 (0.000-0.004) | ref | 0.084 (0.005-0.126) |
| 144 | 0.78 (0.75-0.80) | 0.78 (0.76-0.80) | ref | 0.002 (0.000-0.004) | ref | 0.077 (0.008-0.116) |
| 156 | 0.78 (0.75-0.80) | 0.78 (0.76-0.80) | ref | 0.002 (0.000-0.004) | ref | 0.081 (0.016-0.116) |
| 168 | 0.77 (0.75-0.80) | 0.77 (0.75-0.80) | ref | 0.002 (0.000-0.005) | ref | 0.060 (0.010-0.107) |
| 180 | 0.78 (0.76-0.80) | 0.78 (0.76-0.80) | ref | 0.002 (0.000-0.004) | ref | 0.041 (0.000-0.089) |
| 192 | 0.78 (0.76-0.80) | 0.78 (0.76-0.80) | ref | 0.002 (0.000-0.006) | ref | 0.048 (0.006-0.094) |
| 204 | 0.78 (0.76-0.80) | 0.78 (0.76-0.80) | ref | 0.002 (0.000-0.005) | ref | 0.051 (0.000-0.083) |
| 216 | 0.78 (0.76-0.80) | 0.78 (0.76-0.80) | ref | 0.002 (0.000-0.005) | ref | 0.055 (0.007-0.088) |
| 228 | 0.78 (0.77-0.80) | 0.78 (0.77-0.80) | ref | 0.002 (0.000-0.005) | ref | 0.055 (0.011-0.087) |
| 240 | 0.78 (0.77-0.80) | 0.78 (0.77-0.80) | ref | 0.002 (0.000-0.006) | ref | 0.067 (0.020-0.098) |
| 252 | 0.78 (0.77-0.80) | 0.78 (0.77-0.80) | ref | 0.002 (0.000-0.005) | ref | 0.062 (0.019-0.092) |
| 264 | 0.79 (0.77-0.80) | 0.79 (0.77-0.80) | ref | 0.001 (0.000-0.004) | ref | 0.050 (0.006-0.085) |
| 276 | 0.79 (0.78-0.81) | 0.79 (0.78-0.81) | ref | 0.001 (0.000-0.004) | ref | 0.043 (-0.001-0.076) |
| 288 | 0.79 (0.78-0.81) | 0.79 (0.78-0.81) | ref | 0.001 (0.000-0.004) | ref | 0.045 (0.001-0.08) |
| 300 | 0.79 (0.78-0.80) | 0.79 (0.78-0.81) | ref | 0.001 (0.000-0.004) | ref | 0.044 (0.000-0.069) |
| 312 | 0.80 (0.78-0.81) | 0.80 (0.78-0.81) | ref | 0.001 (0.000-0.004) | ref | 0.041 (-0.002-0.070) |
| 324 | 0.80 (0.78-0.81) | 0.80 (0.79-0.81) | ref | 0.002 (0.000-0.005) | ref | 0.056 (0.016-0.088) |
| 336 | 0.79 (0.78-0.81) | 0.79 (0.78-0.81) | ref | 0.002 (0.000-0.006) | ref | 0.062 (0.019-0.093) |
| 348 | 0.79 (0.77-0.81) | 0.79 (0.77-0.81) | ref | 0.002 (0.000-0.006) | ref | 0.061 (0.015-0.095) |
| 360 | 0.80 (0.77-0.82) | 0.80 (0.77-0.82) | ref | 0.003 (0.000-0.008) | ref | 0.097 (0.030-0.150) |
